# Radiation Risks of Leukemia, Lymphoma and Multiple Myeloma Incidence in the Mayak Cohort: 1948–2004

**DOI:** 10.1371/journal.pone.0162710

**Published:** 2016-09-15

**Authors:** Irina S. Kuznetsova, Elena V. Labutina, Nezahat Hunter

**Affiliations:** 1 Southern Urals Biophysics Institute (SUBI), Ozyorsk, Chelyabinsk Region, Russia; 2 Public Health England (PHE), Centre for Radiation, Chemical and Environmental Hazards (CRCE), Chilton, Oxfordshire, United Kingdom; Kagoshima University Graduate School of Medical and Dental Sciences, JAPAN

## Abstract

Incidence of all types of lymphatic and hematopoietic cancers, including Hodgkin’s lymphoma, non-Hodgkin's lymphoma, multiple myeloma, acute and chronic myeloid leukemia (AML and CML respectively), chronic lymphocytic leukemia (CLL) and other forms of leukemia have been studied in a cohort of 22,373 workers employed at the Mayak Production Association (PA) main facilities during 536,126 person-years of follow-up from the start of employment between 1948 and 1982 to the end of 2004. Risk assessment was performed for both external gamma-radiation and internal alpha-exposure of red bone marrow due to incorporated Pu-239 using Mayak Workers Dosimetry System 2008 taking into account non-radiation factors. The incidence of leukemia excluding CLL showed a non-linear dose response relationship for external gamma exposure with exponential effect modifiers based on time since exposure and age at exposure. Among the major subtypes of leukemia, the excess risk of AML was the highest within the first 2–5 years of external exposure (ERR per Gy: 38.40; 90% CI: 13.92–121.4) and decreased substantially thereafter, but the risks remained statistically significant (ERR per Gy: 2.63; 90% CI: 0.07–12.55). In comparison, excess CML first occurred 5 years after exposure and decreased about 10 years after exposure, although the association was not statistically significant (ERR per Gy: 1.39; 90% CI: -0.22–7.32). The study found no evidence of an association between leukemia and occupational exposure to internal plutonium ERR per Gy 2.13; 90% CI: <0–9.45). There was also no indication of any relationship with either external gamma or internal plutonium radiation exposure for either incidence of Hodgkin or non-Hodgkin lymphoma or multiple myeloma.

## Introduction

Lymphatic and haematopoietic cancers, namely non-Hodgkin lymphoma, (NHL), Hodgkin lymphoma (HL), multiple myeloma (MM), and leukemia have been studied extensively among the Japanese A-bomb survivors, large groups of radiation workers and patients receiving radiotherapy and diagnostic irradiation [1–11]. These studies provide evidence of a relationship between leukemia risk and radiation exposure more than two years after the radiation exposure. Most studies have detected increased risks for acute lymphoblastic leukemia (ALL), acute myeloid leukemia (AML) and chronic myeloid leukemia (CML) subtypes, but not for chronic lymphocytic leukemia (CLL) which appears not to be induced by radiation exposure. For NHL, HL and MM the evidence of a dose-response relationship is lacking due to the limited statistical power [11].

The Registry of radiation workers at the Russian Mayak Production Association (Mayak PA) continues to be one of the most important sources providing information on health effects related to the long term radiation exposure of both genders. These workers, who have now been followed up for more than 50 years, were exposed to both external gamma and internal plutonium. The Registry has already been used to estimate risks of solid cancer mortality and incidence from both external radiation and internal plutonium exposure [10, 12–15]. In addition, risk of leukemia mortality has also been studied [10] and showed increased risk of leukemia other than CLL in relation to external exposure but not for internal plutonium exposure.

The purpose of this study was to analyse radiation risk of lymphatic and haematopoietic cancers incidence in the cohort of Mayak PA workers of main plants. Separate analyses were performed for some types of leukemia and lymphoma and multiple myeloma in relation to external gamma and internal plutonium exposure. Special attention was devoted to Pu exposure factor.

## Materials and Methods

### The study cohort and follow-up

The study methods and cohort descriptions have been published in detail previously [13, 14, 16]. Briefly, the cohort includes 22,373 workers, followed up from the start of employment at one of the main facilities–Reactors, Radiochemical and Plutonium production plants–between 1948 and 1982, up to the end of 2004 (Table 1). Vital status was known for 95% of the workers. At the moment of the end of the follow-up about 59% lived in Ozyorsk, among them 56.5% were alive. (Date of the end of the follow-up is the date of the first cancer registration, or date of death, or date of leaving Ozyorsk, or 31 December 2004, whichever is the earliest.) Most of the workers were first employed at young age (average age was 25 years, 90th percentile 36.7 years). The follow-up period exceeded 25 years for 50% of the cohort members and 25% of workers were followed up to at least 65 years old. A total of 535,877 person-years were accumulated in the study cohort. The information on tobacco-smoking was obtained from medical records and was available for 89.0% of the workers in the cohort.

**Table 1:** Characteristic of Study Cohort by facility.

	Number	Portion (%)
Number of workers	22,373	
Number of females	5,687	25.4
Vital status known (up to Dec 31, 2004)	21,292	95.2
Living at Ozyorsk (up to the end of the follow-up)	13,136	58.7
including alive	7,421	56.5^1^
including died	5,715	43.5^1^
Migrated from Ozyorsk	9,237	41.3
Average age at the date of employment	25.0±7.5	
Average follow-up period	24.0±16.8	
Average attained age	48.9±18.3	

^1^ in proportion to the number of individuals residing in Ozyorsk

As the data on cancer diagnoses stated after the person leaves the town are not available the follow-up period was restricted by the year of departure from Ozyorsk and cancer cases were identified for workers who had lived in Ozyorsk as of the date of diagnosis. The follow-up period started from the year of first employment until the earliest of date of migration from the city, death, diagnosis of the primary malignant neoplasm (excluding non-melanoma skin cancer, NMSC) or the end of follow-up, 31 December 2004.

Information on the diagnoses was obtained from the oncological service’s documents and archive data from medical facilities of the city. Current work contains the diagnoses stated in medical records as it is impossible to review cytological and histological materials. Diagnoses were coded according to the International Classification of Diseases, 9th Revision (ICD-9) [17]. Only the first diagnosed cancer case in each individual was taken into account and follow-up was stopped at the date of first diagnosis of cancer. By the end of 2004, a total of 1,885 malignant neoplasms including 143 cases of all types of hemoblastosis (HB) were identified. Of those, 77 cases were with leukemia (ICD-9: 204–208) and 31 cases with NHL (ICD-9-code: 200, 202.1–202.3, 202.5–202.9), 24 cases with HL (ICD-9: 201) and 11 cases with MM (ICD-9: 203.0, 203.2–203.9). The leukemia cases comprised of 24 cases with acute myeloid leukemia (AML, ICD-9: 205.0), 21 cases with chronic lymphocytic leukemia (CLL, ICD-9: 204.1) and 13 cases with chronic myeloid leukemia (CML, ICD-9:205.1). For other leukemias and unspecified cell types (Other, ICD-9:204.0, 204.2–207.7, 207.9–208.9), there were 19 cases which included one case with acute lymphocytic leukemia (ALL, ICD-9: 204.0).

**Fig 1:**
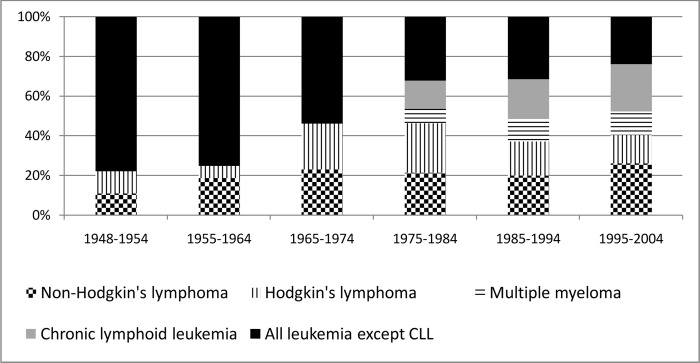
The distribution of diseases by calendar period between 1948–2004.

There are important differences in the frequency of diagnosis of the various types of HB diseases from 1948 to 2004 (Fig 1): 75% of the leukemia cases (84% in men) diagnosed in the first two decades of Mayak PA operation while in the following decades the types of HB diseases diagnosed were almost equally split between lymphoma and myeloma combined (50.8%) and leukemia (49.2%). Among the leukemia subtypes, AML prevailed in the early years of operation of Mayak PA (1948–1964) where it comprised more than 60% of all leukemia cases, but subsequently the proportion of AML decreased rapidly to 19% by the end of follow-up. CLL became the main form of leukemia diagnosed in the 1980s (45%).

### External and internal radiation exposure

The analyses were performed using external and internal dose estimates from Mayak Worker Dosimetry System-2008 (MWDS-2008) [18, 19]. Individual monitoring of external exposure was conducted from the beginning of operation of Mayak and annual dose estimates were available for all the workers of the study cohort. However, for internal exposure, systematic urine monitoring for plutonium (^239^Pu) was not performed until the early 1970s. As a result, plutonium dose estimates are available for only 6,441 workers (38% of 16,995 workers) in the radiochemical and plutonium plants. A small number of workers (260) at the reactor plant) were monitored for plutonium exposure, but the remaining reactor workers (23% of the whole cohort) who were not monitored were considered to have zero plutonium dose. For those workers who were potentially exposed to plutonium, but had no direct measurements, two surrogate categories have been created: high and low category. The categories were defined on the basis of occupational history of each person and values of Pu body burden and accumulated organ dose among Pu monitored workers with the same history. For the purposes of the current paper, the high category included workers first employed at the plutonium plant between 1948 and 1953 and low–all other workers. Mean values of accumulated Pu dose to red bone marrow among monitored workers whose professional history corresponds to the high category made 0.9 Gy, and low– 0.1 Gy.

The absorbed dose to red bone marrow was used in analysis as HB diseases mainly originate in the bone marrow. This dose comprised two parts; external gamma radiation dose and internal alpha radiation dose following the inhalation of plutonium. The mean cumulative external gamma-dose to red bone marrow for the whole cohort was 0.39 Gy (0.41 Gy for men and 0.33 Gy for women) while 90% of the workers received doses below 1.15 Gy. However, the average cumulative external bone marrow dose for leukemia cases exceeded the average in the cohort overall (0.89 Gy). Among the plutonium-exposed cohort members, the cumulative internal doses were generally low; the median dose was 0.01 Gy and only a small fraction of the cohort (10%) accumulated doses exceeding 0.09 Gy.

## Statistical Methods

Poisson regression was used to test for an association between the incidence of HB diseases and exposure to both external gamma radiation and internal alpha radiation to red bone marrow. The statistical methods applied here were similar to those used previously in the analysis of this cohort [13, 14]. Briefly, for each worker, person-years at risk were accumulated over time from the date of first employment at one of the main plants of Mayak PA between 1948 and 1982 up to the end of follow-up either on the date of first cancer registration, or date of death, or date of leaving Ozyorsk, or 31 December 2004, whichever was the earliest. Tabulations of person years and the number of cases were created using DATAB (a module of Epicure [20]) and were described in detail previously [13, 14]. The analyses were carried out with external and internal doses lagged by 2 years for all disease categories considered. A full definition of all variables used in the study is presented in S1 Table.

The model used here is as follows: b_0_ ∙(1+ERR), where b_0_ is the background incidence rate in the absence of radiation exposure and depends on various factors that can affect risks of HB diseases such as attained age, gender etc. and on the excess relative risk (ERR) per unit dose (Gy). The investigation of the dependence of disease risk on external and internal dose was conducted by calculating relative risk estimates or excess relative risk coefficients for one type of exposure and stratified by another. The shape of dose-response was also investigated comparing the fit of a linear dose response model to that of non-linear quadratic (Q) and linear-quadratic (LQ) models. Sensitivity analyses were also performed excluding workers who had received external dose exceeding 1Gy. Differences in the ERR estimates were assessed across levels of modifying factors as well as a continuous log linear function of each modifiers (gender, attained age, age at first exposure and time since exposure). The potential modifying effect of time since exposure on the relationship between HB risk and external dose was further investigated by looking at the effect of partitioning the total cumulative dose into temporal windows. The following model was fitted:

$$\lambda =e^{Z_{i}}∙[1+\beta_{2\_5}∙{dose}_{external,2\_5}+\beta_{5\_10}∙{dose}_{external,5\_10}+\beta_{10+}∙{dose}_{external,10+}]$$ (1)

with dose_external,2–5_, dose_external,5–10_ and dose_external,10+_ defining the exposure incurred 2–5, 5–10 and 10 or more years prior to diagnosis and Z_i_−the set of non-radiation factors used to describe background rate.

The likelihood ratio test (LRT) was conducted to assess whether the linear or quadratic models provide a statistically significant improvement in fit, i.e. the difference in deviance between models was compared with the Chi-square distribution on the appropriate number of degree of freedom. Two-sided p-values were obtained for all statistical tests along with 90% confidence intervals (CI) which were computed using profile likelihood-based methods in Epicure [20].

## Results

As in previous analyses of the Mayak cohort [12–15], this study used the parametric approach for adjusting background rates. The same best fitting model for the baseline risk was used for all disease categories. It consisted of gender-specific linear and linear-quadratic functions of the logarithm of attained age. Additional adjustment of the baseline model for smoking and year of birth did not improve the model significantly. However, among workers who were born after 1915 the baseline rates of other leukemia as a group (RR = 0.28; 90% CI: 0.11; 0.76) were significantly lower than among those born before 1915. There was no evidence of a significant difference between the baseline rates for any type of HB diseases for monitored and unmonitored plutonium workers.

### Internal exposure due to ^239^Pu

Among the 77 cases of leukemia, 47 were monitored for plutonium exposure (7 cases among reactor workers who have zero internal dose). Within the leukemia subtypes, 12 of 24 AML cases, 8 of 13 CML cases and 16 of 21 CLL cases were among monitored plutonium workers. Among the 55 lymphoma cases, 29 were in monitored workers as well as all 11 multiple myeloma cases.

Among unmonitored workers who had the potential to be exposed to plutonium, there was no evidence of a statistically significant effect for the two surrogate categories for any disease groupings. Hence, the further analysis of internal radiation exposure was restricted to those workers to have been monitored with plutonium dose estimates. Table 2 shows the estimates of relative risk by plutonium dose category and the ERR/Gy for the linear dose response model for various diseases. The ERR/Gy for all leukemia was positive, but not statistically significant (p = 0.24). When CLL was excluded from all leukemia, the ERR/Gy remained positive, but again not statistically significantly raised (p = 0.15). Among the leukemia subtypes, the trend was only statistically significantly increased for AML (ERR/Gy = 13.75; 90% CI: <0; 71.46, p = 0.03), but this result is heavily reliant on one case of a worker whose internal dose estimate was 1.57 Gy. When this worker was excluded from the data, the trend estimate was not significant (p>0.5). There was no statistically significant trend for all lymphoma or multiple myeloma (Table 2).

**Table 2:** Estimated relative risk (RR) for lymphatic and haematopoietic cancers by category of cumulative internal plutonium dose to bone marrow and the ERR/Gy among Mayak workers with internal dose^1^.

Internal dose category to bone marrow	All lymphomas		Multiple myeloma		AML		CML		CLL		Other leukemias		All leukemias		All leukemias excluding CLL	
	No.	RR	No.	RR	No.	RR	No.	RR	No.	RR	No.	RR	No.	RR	No.	RR
<0.005*	16	1	8	1	5	1	2	1	5	1	5	1	17	1	12	1
0.005+	13	1.40	3	0.31	6	2.12	6	8.54	11	1.69	7	1.57	30	2.23	19	2.51
Total No. (Female)	29 (3)		11 (4)		11 (2)		8 (1)		16 (7)		12 (4)		47 (14)		31 (7)	
ERR/Gy	3.60		0.03		**13.75**		2.88		-0.12		-		2.13		3.63	
90% CI	(<0; 15.17)		(NA; NA)		(NA; 71.46)		(NA; NA)		(NA; NA)				(<0; 9.45)		(<0; 15.85)	
P-value	0.16		>0.50		**0.03**		>0.50		>0.50		-		0.24		0.15	

^1^ Adjusted for external bone marrow dose.

*: reference category;.

No: number of cases; AML: acute myeloid leukemia; CML: chronic myeloid leukemia; CLL: chronic lymphoid leukemia NA: likelihood confidence intervals cannot be computed because likelihood is flat; **Bold**: statistically significant

### External exposure

The analysis of the incidence risks in relation to external red bone marrow dose showed that the relative risks increased consistently with increasing dose using a 2-year lag for all leukemia and all leukemia excluding CLL (Table 3). The ERR/Gy estimate for all leukemia was statistically significant after adjusting for internal dose (1.90; 90% CI: 0.84; 3.93). The estimate increased to 3.46 (90% CI: 1.57; 7.65) and remained statistically significant when CLL was excluded from all leukemia (Table 3).

**Table 3:** Estimated relative risk (RR) for lymphatic and haematopoietic cancers by category of cumulative external dose to bone marrow and ERR/Gy among Mayak workers.^1^

External dose category to bone marrow	Non-Hodgkin lymphoma		Hodgkin lymphoma			Multiple myeloma		Leukemia subtype								All leukemia		All leukemia excluding CLL	
								AML		CML		CLL		Other leukemia					
	No.	RR	No.	RR		No.	RR	No.	RR	No.	RR	No.	RR	No.	RR	No.	RR	No.	RR
<0.01	6	1*	7	1*		4**	1*	3	1*	1	1*	1	1*	2	1*	7	1*	6	1*
0.01–0.05	5	0.86	2	0.37				2	1.60	2	2.07	3	2.56	2	1.66	9	1.68	6	1.57
0.05–0.20	4	0.48	8	0.94				1	0.70	3	1.58	5	2.21	6	1.77	15	1.81	10	1.84
0.20–1.00	12	1.12	3	0.27		5	1.79	5	3.43	2	0.86	10	2.92	4	2.04	21	2.08	11	1.70
1.00–2.00	3	0.77	4	1.08		2	1.65	8	20.59	5	5.60	1	0.82	2	4.27	16	4.36	15	6.61
2.00+	1	1.03	0		-	0	-	5	54.77	0	-	1	3.75	3	11.15	9	11.4	8	16.18
Total No. (female)	31 (8)		24 (4)			11 (4)		24 (4)		13 (1)		21 (11)		19 (6)		77 (22)		56 (11)	
ERR/Gy	0.09		-0.02			2.39		**13.23**		1.39		-0.02		0.79		**1.90**		**3.57**	
90% CI	(-1.52; 1.45)		(NA; NA)			(-1.28; 35.47)		(4.25; 49.45)		(-0.22; 7.32)		(NA; NA)		(-0.03; 3.76)		(0.84; 3.93)		(1.55; 8.22)	
P-value	>0.50		>0.50			0.14		**<0.001**		0.12		>0.50		0.12		**<0.001**		**<0.001**	

^1^ Adjusted for internal plutonium bone marrow dose.

No: number of cases; AML: acute myeloid leukemia; CML: chronic myeloid leukemia; CLL: chronic lymphoid leukemia

**: those cases exposed <0.2Gy

**Bold**: statistically significant; *Italic*: borderline statistically significance; NA: Not available because likelihood confidence intervals cannot be computed because likelihood is flat

*: reference category.

Among the leukemia subtypes, there was a statistically significant increasing trend in AML with increasing external dose with and without adjustment for internal dose: (ERR/Gy = 13.23, 90% CI: 4.25; 49.45), (ERR/Gy = 13.76, 90% CI: 4.45; 50.79), respectively. For CML there was borderline evidence of an increasing trend in the risk with increasing external dose (ERR/Gy = 1.83, 90% CI: 0.13; 8.88, p = 0.06), but after adjusting for internal dose the estimate was reduced and the trend was no longer statistically significant (ERR/Gy = 1.39, 90% CI: -0.22; 7.32, p = 0.12). There was no excess risk of CLL (p>0.5), other leukemia (p = 0.12), NHL (p>0.5), HL (p>0.5) or multiple myeloma (p = 0.14) using a 2-year lagged doses and the findings did not change when a 10-year lag period was used (not shown here).

The potential non-linearity of the external bone marrow dose-response effect was studied for all leukemia as a single group and for AML and CML leukemia subtypes without adjustment for internal dose, but the findings changed only a little when adjustment for internal bone marrow dose was included. For all leukemias combined, leukemia excluding CLL and for the AML subtype, there was no significant deviation from linearity compared to the LQ model (p = 0.20, p = 0.11 and p = 0.06, respectively) and a pure Q model fitted slightly better than that linear model, but no worse than the linear-quadratic model (p>0.5). For CML, all three models fit the data equally well (p>0.5). Detail for model comparison was presented in Table 4 in supplementary document.

**Table 4:** Model comparison and parameter estimates with 90% CI for the linear and non-linear models for all leukemia and leukemia subtypes in relation to external exposure.

	All leukemias combined	Leukemia excluding CLL	Acute myeloid leukemia		Chronic myeloid leukemia
*Linear model*					
Linear effect	1.89 (0.89; 3.74)	3.46 (1.57; 7.65)	13.60 (4.45; 50.79)	1.83 (0.13; 8.88)	
Deviance change* (P-value)	1.67(p = 0.20)	2.54(p = 0.11)	3.59(p = 0.06)	0.06(p > 0.5)	
*Quadratic model*					
Quadratic term	0.82 (0.40; 1.48)	1.53 (0.75; 2.92)	7.56 (2.69; 23.3)	0.98 (0.08; 3.64)	
Deviance change* (P-value)	0.30 (p>0.5)	0.26 (p>0.5)	0.19 (p>0.5)	0.07 (p>0.5)	
*Linear-quadratic model*					
Linear term	0.53 (-0.84; 2.89)	0.75 (-1.25; 4.76)	2.09 (-4.24; 22.58)	0.91 (-3.38; 6.46)	
Quadratic term	0.63 (-0.19; 1.46)	1.29 (-0.05; 2.83)	7.04 (0.95; 23.64)	0.53(-1.45; 3.78)	

*difference in deviance relative to the linear-quadratic model

When the dataset was restricted to those workers with less than 1 Gy external bone marrow dose, there was no indication of any association with external exposure for all leukemia excluding CLL (p>0.5) and the ERR/Gy was negative. A statistically significant increase of leukemia excluding CLL was found when the accumulated dose was restricted to 1.5 Gy (ERR/Gy = 1.40; 90% CI: 0.23; 3.88) based on a linear model.

### External exposure and modifying factors

Table 5 presents the ERR/Gy for leukemia according to the modifying factors of interest including attained age, age at first gamma-exposure, time since exposure and gender. The ERR/Gy decreased with increasing attained age for leukemia excluding CLL and CML but neither effect was statistically significant. Statistically significant differences were observed for age at first external exposure for AML (p<0.001) and for leukemia excluding CLL although this difference was of borderline significance (p = 0.06). No significant difference was found for CML (p>0.5). There were no statistically significant differences in risk with gender for leukemia excluding CLL or in AML, although the risks were higher in males than females.

**Table 5:** Factors modifying the relationship between cumulative external dose to bone marrow and leukemia incidence risk among Mayak PA workers.^1^

	AML	CML	All leukemias excluding CLL
	ERR/Gy (90% CI, n)	ERR/Gy (90% CI, n)	ERR/Gy (90% CI, n)
*Model 1*: ERR_exdose_ = β_i_dose			
Attained age:			
<55	12.43 (4.05; 45.23, 17)	3.17 (0.41; 14.87, 9)	4.17 (1.94; 8.79, 32)
55+	61.32 (<0; 505, 7)	0.27 (<0; 5.51, 4)	1.36 (0.08; 5.24, 24)
P-value*	0.16	0.24	0.16
Age at first gamma-exposure**:			
<20	<0 (1.17; 20.7, 1)	1.67 (NA; NA, 3)	1.74 (<0; 9.47, 4)
20–29	14.25 (3.81; 66.74, 12)	0.45 (NA; NA, 8)	3.43 (1.34; 8.85, 28)
30–39	113.20 (28.03; 611.40, 10)	- (NA; NA, 0)	11.75 (4.44; 31.86, 16)
40 +	- (NA; NA, 0)	<0 (NA; NA, 2)	6.821 (0.41; 27.77, 5)
P-value*	0.001	>0.5	0.06
Gender effect:			
Male	12.480 (3.56; 53.22, 20)	2.33 (0.24; 12.56, 12)	4.98 (1.98; 13.74, 45)
Female	14.09 (<0; 2.6∙10^4^, 4)	NA (NA; NA, 1)	1.32 (-0.03; 6.07, 11)
P-value*	>0.5	-	0.23
*Model 2*:ERR_ed_ = βdose^2^ x exp [α_tsx_ (tse)+ λ_1_(afe) + λ_2_^2^ (afe)] **			
ERR/Gy^2^ (β)	0.60 (0.08; 2.87)	^-^	0.52 (0.16; 1.26)
Time since exposure: Linear- (α_tsx_)	-1.88 (-2.62; -1.11)	-	-1.91(-2.44; -1.42)
Age at first exposure: Linear-(λ_1_)	-	-	0.22 (0.04; 0.44)
Quadratic-(λ_2_^2^)		-	-0.02 (-0.05; -0.01)
*Model 3a*: ERR = β_2–5_ dose_external,2–5_ + β_5+_ dose_external,5+_ *and Model 3b*: ERR = β_5–10_ dose_external,5–10_ + β_10+_ dose_external,10+_			
	Time since exposure (years)		
	2–5	5–10	10+
Leukemia-CLL (*Model 3a*)	22.62 (10.87; 47.82)	0.60 (-0.11; 2.16)	
AML (*Model 3a*)	38.40 (13.92; 121.4)	2.63 (0.07; 12.55)	
CML (*Model 3b*)	7.86 (1.06; 34.67)		0.86 (<0; 6.14)

^1^ Estimates are not adjusted for internal dose

* Test of homogeneity of the ERR/Gy across categories

**: The estimates are based on positive accumulated gamma-dose only; tse = log(tse/25) and age at first exposure afe = agex-25; NA: Not available because the likelihood is flat

The analysis of non-linearity in the dose response, excluding modifying factors, showed that a pure quadratic model described the trend for leukemia excluding CLL as well as the LQ model. Adding time since exposure and age at first exposure modifying factors as continuous variables substantially changed the ERR estimates. With these modifiers, the Q dose-response model described the data better than the LQ and linear models. Thus the preferred ERR model is quadratic in dose with a log-linear function for time since exposure and a log linear-quadratic function of age at first exposure (Table 5); the ERR at 1 Gy was found to be 0.52 (90% CI: 0.16; 1.26) at 25 years after exposure and age at first exposure of 25 years (Table 5). For AML, time since exposure was found to be the only statistically significant modifying factor (p<0.001), and hence, the preferred model for AML consists of a quadratic function of external dose with a log linear time since exposure. Based on this model, the ERR at 1Gy was 0.60 (90% CI: 0.08; 2.87) at 25 years since first exposure. For CML, none of the effect modifiers modified the ERR for external dose significantly.

Further analysis of time since exposure windows showed that the ERR/Gy for leukemia excluding CLL and for the AML subtype varied significantly with different exposure periods and gave a higher risk per dose unit for more recent exposure than for earlier exposures (Table 5). For CML, however, we considered only exposure in the periods 5–10 years and 10 years or more because there were no CML cases during the first five years of exposure.

## Discussion

This study has shown a statistically significant increasing trend in the incidence of leukemia and leukemia excluding CLL with total external occupational radiation dose to bone marrow irrespective of whether adjustment was made for internal bone marrow doses. Cases with external doses to bone marrow exceeding 1Gy made the main contribution to the association between the risk and dose. Based on a linear dose-response model, the ERR at 1 Gy for leukemia (excluding CLL) was 3.46 (1.57; 7.65). However, this study showed that a pure quadratic model with appropriate effect modifiers described the dose-response relationship better than a linear model. Based on this model, the ERR at 1 Gy for leukemia (excluding CLL) was 0.52 (90% CI: 0.16; 1.26). The previous study examining leukemia mortality in this cohort [10] found a statistically significant trend (ERR/Gy = 0.99; 90% CI: 0.45; 2.12) for leukemia (excluding CLL) which lies midway between the two incidence estimates with and without effect modifiers. The quantitative differences between the results in this study and those obtained from the earlier studies are partly explained by the use of a revised dosimetry system (MWDS-2008) instead of uncorrected dose estimates derived from archival records at Mayak PA. Moreover, incidence analysis was based on a shortened period of follow up in comparison to mortality analysis as follow up was terminated at the moment of migration of an individual from the city. This fact is connected with the inability of collecting data on malignant neoplasms’ incidence for those individuals that had migrated from Ozyorsk. Average age at the moment of migration made 32.2 years while Ozyorsk residents were followed up to 60.7 years averagely. Accumulated doses of gamma-exposure for migrants and residents do not differ substantially and make in average 0.40 and 0.38 Gy respectively. There was also no evidence of significant non-linearity of the dose response curve in the leukemia mortality data, while in the current study a pure quadratic model was found to describe the dose response relationship better than a linear model when the effect modifiers were used.

This study showed no indication of any association with plutonium exposure for all leukemia or leukemia excluding CLL. There was also no indication of a statistical significant increased risk for unmonitored workers from the most hazardous plutonium facilities in the first years of Mayak PA operation. These findings agree with those from the mortality study, which also reported no indication of any effect of internal plutonium exposure on the risk of leukemia excluding CLL [10]. Among leukemia subtypes, this study, did find a statistically significant dose-response relationship between AML and internal exposure, irrespective of whether adjustment was made for external exposure. This result was driven by a case with plutonium bone marrow dose exceeding 1 Gy and the trend lost its significance after this case was excluded (p>0.5).

Among risk modifiers, time since exposure was found to have a major effect on the risk of leukemia excluding CLL incidence in relation to external exposure; the ERR/Gy decreased significantly with increasing time since exposure. Cases were most frequently diagnosed during the first 2–5 years after initial exposure and the risk estimate for leukemia excluding CLL was 38 times higher in this period than in later periods. The previous mortality study reported a 15 fold difference in the ERR risk between doses received 3–5 years prior to diagnosis compared to those received more than 5 years before[10].

Among leukemia subtypes, a strong statistically significant dose response relationship was observed between external exposure and AML, but not for CML, CLL or other leukemia subtypes. A pure quadratic dose response model also provided a statistically significant better fit than a linear model for AML and time since the first exposure was found to be the only significant effect modifier (ERR at 1 Gy = 0.60, 90% CI: 0.08; 2.87 at 25 years since the first exposure). This study found no evidence of a relationship between either external or internal exposure and the incidence of Hodgkin, non-Hodgkin lymphoma, and multiple myeloma. These findings cannot be compared with the mortality data as this is the first study to present the results for all lymphatic and hematopoietic cancers in the Mayak worker cohort.

### Comparison with other studies

A wide range of studies using mortality and incidence data have shown that leukemia can be caused by exposure to both acute and chronic external gamma radiation [1–11]. However neither these studies nor this Mayak cohort have also demonstrated evidence of radiation effects on CLL rates.

A large-scale study of International Radiation Workers in the Nuclear Industry [1] found that the external radiation risk was elevated for leukemia excluding CLL but not significant (ERR = 1.93, 90% CI: <0; 7.14, p = 0.13). In addition, the 3^rd^ analysis of the National Registry for Radiation Workers (NRRW) in the UK strengthened the evidence for increased leukemia risks from protracted low-dose external exposure and the study reported a linear statistically significant increasing trend in relation to external dose for leukemia excluding CLL, but found a lack of evidence for temporal variations [5, 6]. Despite the small numbers of cases in the current study, our results for leukemia excluding CLL show good agreement with the NRRW study (Table 6).

**Table 6:** Comparison of the Excess Relative Risk estimates of leukemia excluding CLL and subtypes incidence due to exposure to external radiation.

Study	Range of red bone marrow doses (Gy) (mean dose)	Study Population	Leukemia excluding CLL	Acute myeloid leukemia	Chronic myeloid leukemia
			ERR at 1 Gy (90% CI, n)		
*Mayak PA*(Current study)	0–5.26 (0.33)	22,373	0.52(0.16; 1.26, 56)#	0.60(0.08; 2.87, 24)#	1.83(0.13; 8.88, 13)+ Θ
			3.46(1.57; 7.65, 56)**+**		
*3*^*rd*^ *NRRW*	0–0.1+ (0.025)**	174,541	1.78(0.17; 4.36, 234)*	0.62(-1.45; 4.83, 109)	4.08(0.88; 11.2, 59)
Japanese A-bomb survivors*	0–4.54 (0.64)	113,011	2.78(1.84; 4.01, 312) ^a^	1.1(0.53; 2.08, 176)^b^	5.24(1.92; 11.8, 63)^c^
					1.17(-0.10; 4.71, 12)^d^

#: Based on a pure quadratic dose-response with effect modifications (time since exposure, age at exposure)

**+**: Based on a linear dose-response

Θ: p = 0.06

*: 95% CI

**: Doses are based on body surface

a: A linear dose response model, ERR/Gy at age 60 after exposure at age 25

b: A pure quadratic dose response model, ERR/Gy at age 70 after exposure at age 30+

c: A linear dose response, ERR/Gy at age 55 after exposure at age 25 in Hiroshima.

d: A linear dose response, ERR/Gy at age 55 after exposure at age 25 in Nagasaki

**Fig 2:**
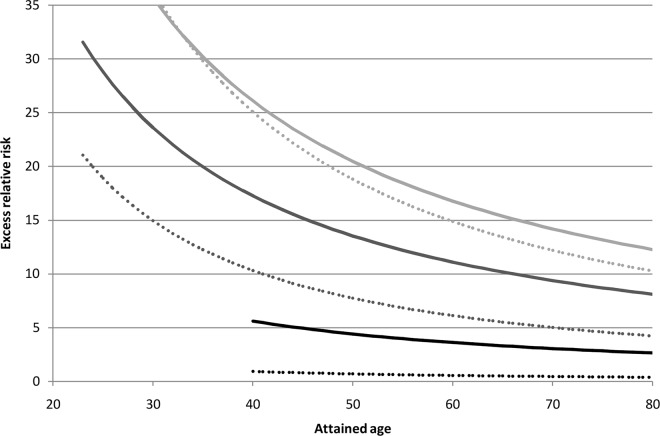
Excess relative risk of leukemia excluding CLL due to accumulated external gamma-dose of 1 Gy in relation to time since exposure and attained age. Solid lines: LSS cohort (excess risk parameters estimates from Table 4 of [3]); dotted lines: Mayak workers cohort; light grey: 3 years since exposure; dark grey: 5 years since exposure; black lines: 20 years since exposure.

A comparison of our results with those based on the LSS cohort is shown in Fig 2. In both cases the ERR model for leukemia excluding CLL demonstrated a non-linear dependency on external gamma-dose taking into account risk modifying factors time since exposure and attained age. The results are comparable for the first three years since exposure, but differ for exposures received in more distant periods (Fig 2).

The LSS study reported a significant non-linear dose-response relationship for leukemia excluding CLL incidence which was driven mainly by cases of acute myeloid leukemia (AML). While in the NRRW study CML was the most strongly associated with external radiation exposure, but for AML the ERR was greater than zero though the trend was not statistically significant. In the Mayak PA cohort, AML was the most frequently observed type of leukemia associated with external exposure with the risks decreasing significantly with increasing time since exposure. These findings partly agree with those from the LSS [3], which reported a significant curvature of the dose-response trend and showed that time since exposure and age at exposure were significant risk modifiers. However, unlike the Mayak data, in the LSS cohort radiation-induced excess AML cases were observed throughout the follow-up period (up to 55 years after the bombings). Despite the difference in the pattern of AML risk, the central estimates in both studies are consistent. In addition, the estimate of risk for AML in this study is the same as that in the 3rd NRRW (Table 6). For CML, a weak linear relationship with external dose (p = 0.06) was observed in the Mayak PA cohort, but there were no CML cases during the first 5 years after external exposure, while in both the LSS and the NRRW 3^rd^ analysis CML cases generally started to arise directly after exposure. The ERR/Gy for CML in the Mayak cohort was found to be lower than the estimate from the NRRW analysis and the LSS Hiroshima specific estimate, but higher than the LSS Nagasaki specific estimate although the estimates did not differ significantly (Table 6).

Studies of populations exposed to radiation either occupationally or for other reasons have shown ambiguous results in relation to the risks of lymphoma and multiple myeloma [1, 3, 5–8, 11]. An analysis of radiation workers in the USA has shown increased risk of non-Hodgkin lymphoma (NHL) mortality with external exposure [2], while in the 3^rd^ NRRW in the UK workers, no association was found for the NHL mortality and only a weak association for NHL incidence [5, 6]. In addition, a significantly increased incidence of multiple myeloma with external dose was reported in the 3^rd^ NRRW study although the authors cautioned that the reliability of the result was low, based on few cases with relatively high doses. In contrast, the current study found no association between either Hodgkin or non-Hodgkin lymphoma or multiple myeloma incidence and external exposure. Similar results were also obtained in the LSS although there was some evidence of increased incidence of NHL with external dose among males. The majority of published studies used a 10-year lag period to assess the risk of lymphoma and multiple myeloma, while in the current study a 2-year lag period was used for lymphoma and multiple myeloma. The first case of lymphoma in the cohort of Mayak PA workers was registered mostly within two years after the first employment, but for multiple myeloma the first case was registered after 18 years. When 10-year lag period was used, the resulting risks estimated did not change the overall conclusions regarding lymphoma and multiple myeloma risk. It should also be noted that using the dose to red bone marrow in the study of radiation-induced risk of lymphomas did not distort significantly the results related to external gamma-exposure due to similarities of dose estimates for different organs and tissues.

The analysis of the relation between the level of internal exposure from 239Pu and lymphatic and haematopoietic cancers incidence risk based on internal dose estimates for red bone marrow was performed for the first time in this cohort. No statistically significant internal dose trend was detected for any type of HB diseases. The previous study of leukemia mortality among cohort studies of people exposed to internal alpha radiation [10] provided no indication of a significant effect of plutonium exposure (p>0.5).

Much effort has been made to produce the best quality dose estimates for internal plutonium exposure for the members of the Mayak cohort in recent years. However, a limitation of this study, in particular the proportion of unknown plutonium dose estimates among workers in the early years, has contributed to uncertainties about the risk assessment of internal exposure. The selection of target organ for internal dosimetry may also lead uncertainty in various forms of lymphoma. Furthermore, unknown incidence data among the migrants and the small number of observed cases reduce the statistical power of the study. Despite these issues, the study has provided evidence of an increased risk of leukemia for external exposure and no association between plutonium exposure and either leukemia or lymphoma or multiple myeloma.

## Supporting Information

S1 Table Description of variables used the study. (DOCX)
