# Supplementary material for: Radiation Risks of Leukemia, Lymphoma and Multiple Myeloma Incidence in the Mayak Cohort: 1948–2004
Source: PLoS One. 2016 Sep 15;11(9):e0162710. doi: 10.1371/journal.pone.0162710 (PMC5025099; doi:10.1371/journal.pone.0162710)
Supplement: S1 Table — (DOCX) [file pone.0162710.s001.docx]

S1 Table. Description of variables used the study

| **Name of variable** | **Description of variable** | |
| --- | --- | --- |
| Sex | 1:Males | 2:Females |
| Bcohort | Birth cohort categories  1:<1910  2:1910-1919  3:1920-1929 | 4:1930-1939  5:1940-1949  6:1950-1959  7:1960+ |
| YrEmp | First Year of Employment:  1:1947-1953  2:1954-1958  3:1959-1972 | 4:1973-1982  5:1983-2002 |
| AgePuCat | Age at first Plutonium Exposure  1:<20  2:20-25 | 3:25-30  4:30+ |
| AgeGammacat | Age at first External Exposure  1:<20  2:20-25 | 3:25-30  4:30+ |
| Pupot | Plutonium surrogate categories:  1: reactor workers hired in any period  2: main plutonium plant, hired 1964–1972, or auxiliary plutonium plant hired 1959–1972 or radiochemical plant, hired 1954–1972  3: plutonium plant, hired 1959–1963 or auxiliary plutonium plant, hired 1950–1958 or radiochemical plant, hired 1948–1953  4: plutonium plant, hired 1954–1958 or auxiliary plutonium plant, hired 1948–1949  5: main departments of the plutonium plant hired 1950–1953  6: main departments of the plutonium plant hired 1948–1949 | |
| Agecat | Attained age categories  1: 15–20  2: 20–25  3: 25–30  4: 30–35  5: 35–40  6: 40–45  7: 45–50 | 8: 50–55  9: 55–60  10: 60–65  11: 65–70  12: 70–75  13: 75–80  14: 80–85  15: 85+ |
| Ctime | Calendar time categories  1: 1947-1950  2: 1951-1955  3: 1956-1960  4: 1961-1965  5: 1966-1970  6: 1971–1975 | 7: 1976–1980  8: 1981–1985  9: 1986-1990  10: 1991–1995  11: 1996–2000  12: 2001–2005  13:2006-2008 |
| Pumon2 | Plutonium monitoring status lagged 2 years  0:unmonitored until 2 years after initial monitoring date  1:monitored | |
| Mig | Migration status  0:while resident in Ozersk  1:after migration from Ozersk | |
| Gamma2cat | Cumulative external RBM dose categories lagged 2 years  1: 0–5 mGy  2: 5–10  3: 10–20  4: 20–50  5: 50–100  6: 100–150 | 7: 150–200  8: 200–300  9: 300–500  10: 500–750  11: 750–1000  12: 1000–1500  13: 1500–2000  14: 2000–3000  15: 3000+ |
| Pu2cat | Cumulative plutonium RBM dose categories lagged 2 years  1: 0  2: >0-1 mGy  3: 1-2  4: 2-5  5: 5-10  6: 10-20  7: 20-50  8: 50–100  9: 100–200 | 10: 200–500  11: 500–1000  12: 1000–1500  13: 1500–2000  14: 2000–3000  15: 3000-5000  16: 5000-7500  17: 7500-10000  18: 10000-15000  19: 15000 + |
